# Treatment of Provoked Vulvodynia in a Swedish cohort using desensitization exercises and cognitive behavioral therapy

**DOI:** 10.1186/s12905-015-0265-3

**Published:** 2015-11-25

**Authors:** Suzanne Lindström, Linda J. Kvist

**Affiliations:** Sexology Department, Najaden Midwifery Clinic, Drottninggatan 7, 252 21 Helsingborg, Sweden; Department of Obstetrics and Gynecology, Helsingborgs Hospital, 25187 Helsingborg, Sweden; Department of Health Sciences, Faculty of Medicine, Lund University, 22100 Lund, Sweden

**Keywords:** PVD, CBT, Desensitization exercises, Combination treatment, MFSQ, HADS

## Abstract

**Background:**

Problems related to pain during vaginal penetration are complex and the etiology is multi-factorial. It was the aim of the present study to measure whether treatment using desensitization exercises and cognitive behavioral therapy (CBT) for women with provoked vulvodynia (PVD) could increase sexual interest, sexual satisfaction and response whilst decreasing experiences of sexual pain.

**Methods and outcome measures:**

Sixty women suffering from PVD were treated during a 10-week period with a combination of mucosal desensitization and pelvic floor exercises and CBT. The McCoy Female Sexuality Questionnaire (MFSQ) was used to measure efficacy of the treatment. The Hospital Anxiety and Depression Scale (HADS) was used to measure psychological distress. The primary outcome measurements were changes in scores for the MFSQ and changes in individual items on the MFSQ directly after treatment completion. Secondary outcome measurements were changes in the MFSQ items 6 months after treatment and changes in HADS sub-scales 6 months after treatment. Statistical comparisons of answers to the MFSQ were carried out using the Wilcoxon signed rank test (paired). Validity of the MFSQ in this study was measured by testing one global question about sexuality and total scores on MFSQ using Spearman’s correlation test.

**Results:**

Study participants reported a statistically significant increase in sexual fantasies, increased sexual pleasure, excitement and vaginal lubrication after treatment was completed. PVD occurred less often which resulted in significantly less avoidance of sexual intercourse, increased frequency of masturbation and intercourse. All improvements were sustained at 6 months after treatment ended. Two questions showed no significant changes, these pertained to the individual’s contentment with her partner as a lover and a friend. The anxiety sub-scale of the HADS showed a significantly decreased level of anxiety at 6 months follow-up but no change in the scores on the depression sub-scale.

**Conclusion:**

Treatment for PVD using desensitization exercises and cognitive behavioral therapy significantly improved sexual interest, response and activity and decreased the experience of pain. Larger studies and RCTs are required in order to draw conclusions about treatment and long term effects should be studied. Partners should be encouraged to participate in treatment regimes.

**Trial registration:**

The study is registered with ISRCTN registry, ID ISRCTN40416405.

## Background

Provoked vulvodynia (PVD) may seriously affect quality of life in women who suffer from the condition [1]. The problem is complex and because etiology has been scantily studied, national and international consensus on guidelines for treatment, have yet to be reached [2]. Internationally, statistics regarding prevalence of painful sexual intercourse or painful attempted intercourse vary between 14 and 34 % [3]. A large population study was carried out in USA in 2003 and showed that as many as 14 million women in USA suffer from chronic vulval pain during their lifetime [4]. The scientific community uses several different sources in order to define symptoms related to painful sexual intercourse, generally named dyspareunia. The International Society for the Study of Vulvo-vaginal Disease (ISSVD) proposed a nomenclature for the pain syndrome: vulvodynia [5]. In the 2013 version of Diagnostic and Statistical Manual of Mental Disorders (fifth edition), vaginismus and superficial coital pain are defined as Genito-Pelvic Pain/Penetration Disorder and are found under the pain disorder section [6]. In an up-date of guidelines for treatment of vulvodynia, it has been suggested that the classification of vulvodynia depends on the site of pain, whether it is provoked or unprovoked and whether the pain is generalized or localized [2].

Vulvodynia means pain in the vulval area. Some patients have continuous generalized pain in the vulval area, others suffer from more localized pain, provoked by vaginal intercourse or attempts at vaginal intercourse, tampon use and also gynecological examination. Generalized unprovoked vulvodynia is less common. PVD, formerly referred to as vulvar vestibulitis syndrome, is the most common form of vulvodynia in young women [4]. In this article the term PVD will be used.

Women describe PVD as burning stinging, irritation or rawness [2]. The problem often has a multi-factorial background which means that treatment calls for considerable health service resources. PVD causes sexual problems including reduced libido, difficulties in achieving a subjective genital response and maintaining desire during sexual activity, orgasm problems and sexual pain [1]. American researchers showed that as many as 40 % suffered in silence and never approached health care providers for help and of those who did seek help, 60 % consulted three or more doctors without a diagnosis being made [4]. Women who suffer from PVD often have tense pelvic muscles, which have developed as a result of the body’s attempts to protect itself from pain. This may develop further to a partial or complete vaginismus when penetration is attempted [7, 8].

In Sweden PVD is a common problem amongst young women who consult gynaecologists, midwives and general practitioners although they may not state it to be their primary reason for consultation [9–11]. It has been suggested that approximately 13 % of women aged 20 to 29 years suffer from these problems [9]. Research on younger Swedish women between 13 and 22 years of age showed that painful sexual intercourse occurred in 47–49 % of young women who continued to have intercourse despite their painful experiences [10]. Many treatments, both physiological and psychological, have been used to help these women, including acupuncture, tricycle antidepressants, physiotherapy, electromyography (EMG), de-sensibilization exercises, psychotherapy and CBT (Cognitive behavioural therapy) and surgical excision [12–18].

The complex nature of the problem suggests that treatment for PVD requires care which focuses on relationships, thoughts, emotions and knowledge about the body, psyche and behaviour in order to improve sexual capacity and health. The aim of the present study was to examine the effect of a treatment regime offered to women suffering from PVD in southern Sweden.

## Methods

This is a descriptive study of a cohort of women who attended a clinic for sexual problems between 2012 and 2014 and were offered a combination treatment for their PVD which continued for 10 weeks and focused on behavioural, emotional and physical aspects. The treatment used physical and psycho-sexological methods since research has shown the benefits of combinations of this kind [1, 8, 13, 15, 16, 19]. Effects of the treatment regime were measured using the McCoy Female Sexuality Questionnaire (MFSQ) [20]. In an unpublished quality control study, the authors discovered that a substantial number of women who sought help for PVD reported a history of depression and therefore the Hospital Anxiety and Depression Scale (HADS) was also used in the present study [21–23].

### Study participants

A sample size calculation was carried out, based on previous results from a quality control study (unpublished) at the same clinic. Based on the results, we anticipated a 5 % increase in scores on MFSQ after treatment (α = 0.05, β = 0.80) showing a necessary sample size of 58 for a double-sided, pairwise analysis.

### Inclusion and exclusion criteria

Women who were referred to the authorized sexual advisor and trained CBT therapist in this study had first been diagnosed by a doctor. The diagnosis of PVD is typically based on the patient’s self-report of pain at the entrance to the vagina during any attempt at penetration and is confirmed by the cotton- swab test [24]. The women invited to participate had suffered their symptoms for at least 6 months and had (a) severe pain upon vestibular touch or attempted vaginal entry, (b) tenderness to pressure localized within the vulvar- vestibule and (c) vestibular erythema of various degrees. The pain is often located between 4 and 8 o’clock on the introitus, just exterior to the hyminal ring [17]. Women were excluded when they had current infection or diagnosed dermatological disease of the genital area, a diagnosis of acute psychiatric illness or any other major medical problems requiring medical treatment.

### Ethical aspects

This study was approved by the Regional Ethics Committee in Lund, Sweden, (Dnr: 2012/116). PVD is a condition which affects women’s self-esteem [1, 7] and for this reason treatment demands special prudence. The woman’s autonomy must be respected allowing her to be involved in her treatment. Her personal experiences should be carefully listened to, confirmed and never questioned for their accuracy. The cultural, social and psycho-sexual lives of each individual must also be respected. The women were aware that the answers they gave to the questionnaires would be the basis for discussions and decisions regarding their individual treatment. The participants in this study were informed about the therapist’s obligation to professional secrecy. Case notes are protected against access by anyone other than the treatment team. In this study, the referring doctor, the medical secretary and the therapist were the only people with access to the case notes. Information was also given regarding the participant’s right to terminate inclusion in the study at any time, without any effect on the on-going treatment.

### Recruitment and collection of data

When the women came for their first consultation with the therapist they were invited to join the study before the consultation began and were given verbal and written information. Those who were willing were asked to fill in questionnaires on which individual dialogue and treatments would be based. A history questionnaire was based on a questionnaire drawn up by the Swedish Society for Obstetrics and Gynaecology [25] and included questions regarding social situation, when PVD problems began, sexual experiences and preferences, which treatments they had earlier received, previous illnesses relative to the present problem, life style questions and health status. The questionnaire is very extensive and only a small number of variables are reported in this paper. A 12-question version of the MFSQ was used to measure sexual interest, response and activity of participants at three points in time: before commencement of treatment, directly after completion of treatment and at 6 months after treatment. The participants were also asked to fill in the HADS questionnaire to measure psychological distress.

### The treatment

During a 10-week period the women recruited to the study attended ten treatment sessions. These sessions focused on discussions about the woman’s intimate relationship, on improving the individual’s knowledge of her bodily functions, helping her to train her sexual responses and learning to feel sexual pleasure on the same terms as her partner so that both could experience pleasure and no one’s needs dominated. Each session took approximately 60 min. Discussions about the individual woman’s life situation, about couple relationships and about how sexual pain may reflect relationship problems were an integral part of the CBT. During the 10-week period the woman had contact with the same therapist. The therapist herself received regular supervision sessions with a cognitive psychotherapist/psychiatrist. All treatments were carried out by the same therapist and were individualised which meant that some women made quicker progress than others but all were offered the full ten sessions.

The sessions included information and education about dyspareunia, PVD and vaginismus and how pain impacts desire, sexual response and arousal. A presentation was given of the female genital anatomy, using a detailed plastic model and photographs of the vulva and vagina. A gynaecological examination was carried out sensitively and carefully and the woman’s individual pain problems were confirmed. With the aid of a hand mirror the woman showed where her pain was located and explained her sensations of pain. The woman was asked to avoid soap for intimate hygiene and instead to use neutral oil. If the woman lived in a relationship with a partner she was instructed that penile penetration should be avoided until there was improvement in her experience of pain. Goals for treatment were negotiated between the patient and therapist and it was important also to formulate interim goals, such as being able to carry out a vaginal examination or penile penetration without pain or with reduced pain.

The woman was given information about CBT which required some homework in the form of training to re-direct negative thought-pathways. Many women have repressed their thoughts about sexual activity and part of their homework was also to allow these thoughts freedom and to give thought to what they wanted and needed sexually. An important example of this kind of re-direction is in how the woman might express her pain and problems for her partner.

Exercises aimed to desensitize the pain memory response were gradually initiated and included training to feel tension and relaxation in the muscles of the pelvic floor and vagina by insertion into the vagina of one or two of the woman’s own fingers and progressively the partner’s fingers. Rather than avoidance of touch to the painful area the woman was instructed in regular, daily self-examination using a hand mirror, to touch and massage the area with oil and to use acupressure. Use of tampons during menstruation was encouraged. When the woman felt that she had mastered the exercises and if she was living together with a partner she was asked to gradually introduce the partner to partake in the exercises. Results of the homework were discussed each time the woman and therapist met.

At each new session an agenda was formulated by the woman and therapist together and was always initiated with the question “*How have things been since our last meeting*”? The woman was also encouraged to give a synopsis and critique of the previous session. At each session a vaginal examination was carried out with the woman partaking actively by means of a hand mirror. A dialogue was upheld about how the woman’s sex life and relationship was developing. Sex resources such as vibrators, dildos and lubricating gels were introduced progressively if these were acceptable. A discussion ensued about how the homework had progressed and new homework was negotiated. Finally the woman was asked to make a summary of the day’s session and feed-back was given by the therapist.

### Instruments and outcome measurements

Outcome measurements were recorded at three points in time; at the first consultation, after 10 weeks completed treatment and again at 6 months after treatment completion. The MFSQ questionnaire was originally developed in USA and contained 19 items [20]. In international studies the questionnaire has been adjusted from the original 19 to seven, nine or ten items. A version with 12 items was used in the present study. The 12 items concern sexual motivation (contentment with the extent of sexual activity, fantasies, desire and excitement), sexual response (lubrication, frequency of sexual intercourse, orgasm and masturbation), occurrence of dyspareunia and relationship to the partner as a lover and as a friend. Nine of the items were answered on a seven step Likert-type scale where 1 = “never” or “very dissatisfied” and 7 = “very satisfied”, “very pleasurable” or “every time”. Two items about intercourse and masturbation during the preceding month were answered on a six step scale where 1 = “never” and 6 = “more than eight times”. The final item which pertained to avoidance of sexual intercourse because of pain was answered on a five point scale where 1 = “always” and 5 = “never”. For those who had not partaken in sexual activity or did not have a partner, it was possible to answer “not relevant”. The highest total score on the MFSQ used in this study was 80 points.

A global question answered on a visual analogue scale (VAS) was posed each time the MFSQ was used: “*How do you feel about the extent of your sexuality and your sexual life today?(Whether you currently have a partner or not)”.* The end points of the VAS were: 0 = “Very dissatisfied” and 10 = “Very satisfied”. This question was asked in order to measure correlations between the question and scores on the MFSQ in order to provide a measure of validity.

The women were also asked to fill in HADS at the same time points as the MFSQ scale. HADS questionnaire is widely used to measure psychological distress such as anxiety disorders and depression among patients in non-psychiatric hospital clinics [21–23, 26]. HADS is divided into two sub-scales, anxiety and depression. Items are scored on a 4-point Likert scale ranging from 0 to 3. Maximum possible total scores for anxiety are 21 and 21 for depression.

### Statistical analyses

The material generated by the questionnaires was analyzed using Predictive Analytics Software (PASW version 17.0). Descriptive statistics were used to present some answers from the history questionnaire. In order to evaluate changes in the woman’s sexual motivation, response, occurrence of PVD and relationship with her partner before, directly after completion of treatment and at a 6 months follow-up, statistical comparisons of mean scores on the MFSQ (paired samples t-test) and comparisons of answers to the individual items on MFSQ (Wilcoxon signed-rank test) were carried out. Total mean scores for the HADS sub-scales anxiety and depression, were compared between measurements using paired samples t-test since the range of scores was 0–21 for HADS sub-scales. Correlation tests between the global question and the three total MFSQ scores were carried out using Spearman’s correlation test. A *p*-value of ≤0.05 was considered to be significant.

## Results

Between 2012 and 2014 a total of 60 women were treated, median age 27.5 years (range 16–66 years). All of the women gave written consent to partake in the study. Of these women, 59 answered a question regarding duration of symptoms and they reported having symptoms a median of 4 years (range 0.5–22 years). A total of 88.3 % (*n* = 53) were involved in a permanent relationship, 10 % (*n* = 6) were not in a permanent relationship and 1.7 % (*n* = 1) did not answer the question. Table 1 shows information regarding participants’ earlier sexual experiences. There was a statistically significant increase between total mean scores on the MFSQ before and directly after treatment (*p =* <0.01) and these numbers are shown in Table 4. Table 2 shows results of the primary outcome measurement comparing results for the individual MFSQ questions before and directly after treatment.

**Table 1:** Respondents answers to questions included in the history questionnaire regarding earlier sexual experiences

Question and response	*n* (valid %)
Have you ever had good sexual experience?	
“Yes”	46 (78)
Have you ever had bad sexual experience e.g. violence or coercion?	
“Yes”	14 (23.7)
Have you had bad experience of gynaecological examination?	
“Yes”	20 (34.5)
Have you experienced pain which made intercourse impossible?	
“Yes”	37 (62.7)
Have you felt a lack of sexual desire?	
“Yes”	48 (80)
How often have you had intercourse during the preceding year?	
Not had intercourse	15 (26.8)
1–5 times	13 (23.2)
6–10 times	7 (12.5)
11–20 times	6 (10.7)
> 20 times	15 (26.8)
Do you reach orgasm with your partner?	
Never	14 (23.3)
Sometimes	29 (48.3)
Always	17 (28.3)
Do you reach orgasm by masturbation?	
Never	20 (33.3)
Sometimes	16 (26.7)
Always	24 (40)

**Table 2:** Scores for all questions on the MFSQ (mean and standard deviation) and comparison of individual question scores before and directly after completed treatment using Wilcoxon signed rank test (paired)

Question	MFSQ (before treatment)	MFSQ (directly after treatment)	
	Median (range)	Median (range)	*p*-value
1. Are you satisfied with the extent of your sexual life at the present moment? (1 = very dissatisfied, 7 = very satisfied)	2.0 (1–6) *n* = 60	5.0 (1–7) *n* = 59	<0.01* (z −6.50)
2. Approximately how many times, during the last month, have you thought about sex or had sexual fantasies? (1 = never, 7 = >10 times per day)	3.0 (1–6) *n* = 60	4.0 (1–7) *n* = 59	<0.01* (z −5.10)
3. Does sexual activity give you pleasure? (1 = no pleasure at all, 7 = a lot of pleasure)	4.0 (1–7) *n* = 53	6.0 (13–7) *n* = 53	<0.01* (z −5.10)
4. How often do you feel sexually excited or stimulated during sexual activity? (Example: increased pulse, increased vaginal lubrication, skin redness, quicker breathing, increased sensitivity in the skin and erogenous areas). (1 = never, 7 = every time)	4.0 (1–7) *n* = 53	6.0 (2–7) *n* = 54	<0.01* (z −4.51)
5. How often do you achieve orgasm during sexual activity? (For example increased pulse, increased vaginal lubrication, skin redness, quicker breathing, increased sensitivity in the skin and erogenous areas). (1 = never, 7 = every time)	4.0 (1–7) *n* = 53	6.0 (1–7) *n* = 54	<0.01* (z −3.85)
6. How often is vaginal dryness a problem during sexual activity? (1 = every time, 7 = never)	4.0 (1–7) *n* = 54	6.0 (2–7) *n* = 54	<0.01* (z −3.60)
7. How often is sexual intercourse painful? (1 = every time, 7 = never)	5.5 (1–7) *n* = 53	6.0 (2–7) *n* = 53	<0.01* (z −6.01)
8. Are you satisfied with your partner as a lover? (1 = very dissatisfied, 7 = very satisfied)	7.0 (2–7) *n* = 55	6.0 (1–7) *n* = 54	0.75 (z −0.32)
9. Are you satisfied with your partner as a fellow human and friend? (1 = very dissatisfied, 7 = very satisfied)	7.0 (2–7) *n* = 54	7.0 (2–7) *n* = 54	0.06 (z −1.90)
10. How often have you had sexual intercourse during the last month? (1 = never, 6 = >8 times)	2.0 (1–6) *n* = 60	3.0 (1–6) *n* = 59	<0.01* (z −4.51)
11. How often have you satisfied yourself, alone, during the last month? (1 = never, 6 = > 8 times)	1.0 (1–6) *n* = 60	4.0 (1–6) *n* = 59	<0.01* (z −5.90)
12. How often do you avoid intercourse in order to avoid pain? (1 = always, 5 = never)	2.0 (1–5) *n* = 58	4.0 (12–5) *n* = 52	<0.01* (z −5.71)

*Statistically significant increase in scores

Table 3 shows the secondary outcome measurement which is a statistical comparison of the answers to the separate questions on the MFSQ before treatment and 6 months after treatment. Statistically significant increases in scores for 10 of the 12 questions were sustained at 6 months after completion of treatment. The participants showed statistically significant increased satisfaction with the extent of sexual activity, sexual excitement occurred more often, there was an increase in sexual fantasies and increased pleasure in sexual activities. The women reported reduced pain, reduced genital dryness, sexual intercourse was avoided less often and orgasm occurred more frequently. Frequency of masturbation increased. There were no statistically significant changes in the respondents’ satisfaction with their partners as lovers or as fellow humans and friends either directly after treatment or after 6 months. These scores were initially high, as shown in Table 2.

**Table 3:** Comparisson for all questions on the MFSQ (mean and standard deviation) before and 6 months after completed treatment using Wilcoxon signed rank test (paired)

Question	MFSQ (before treatment)	MFSQ (6 months after treatment)	
	Median (range)	Median (range)	*p*-value
1. Are you satisfied with the extent of your sexual life at the present moment? (1 = very dissatisfied, 7 = very satisfied)	2.0 (1–6) * n* = 60	5.0 (1–7) *n* = 60	<0.01* (z −6.71)
2. Approximately how many times, during the last month, have you thought about sex or had sexual fantasies? (1 = never, 7 = >10 times per day)	3.0 (1–6) * n* = 60	4.0 (1.5–7) *n* = 60	<0.01* (z −4.50)
3. Does sexual activity give you pleasure? (1 = no pleasure at all, 7 = a lot of pleasure)	4.0 (1–7) *n* = 53	6.0 (3–7) *n* = 54	<0.01* (z −4.60)
4. How often do you feel sexually excited or stimulated during sexual activity? (1 = never, 7 = every time)	4.0 (1–7) * n* = 53	6.0 (3–7) *n* = 53	<0.01* (z −5.10)
5. How often do you acheive orgasm during sexual activity? (1 = never, 7 = every time)	4.0 (1–7) *n* = 53	5.0 (1–7) *n* = 53	0.01* (z −2.50)
6. How often is vaginal dryness a problem during sexual activity? (1 = every time, 7 = never)	4.0 (1–7) *n* = 54	5.0 (1–7) *n* = 53	<0.01* (z −3.13)
7. How often is sexual intercourse painful? (1 = every time, 7 = never)	5.5 (1–7) *n* = 53	6.0 (1–7) *n* = 53	<0.01* (z −5.90)
8. Are you satisfied with your partner as a lover? (1 = very dissatisfied, 7 = very satisfied)	7.0 (2–7) *n* = 55	6.0 (2–7) *n* = 51	0.61 (z −0.61)
9. Are you satisfied with your partner as a fellow human and friend? (1 = very dissatisfied, 7 = very satisfied)	7.0 (2–7) *n* = 54	7.0 (1–7) *n* = 53	0.10 (z −1.71)
10. How often have you had sexual intercourse during the last month? (1 = never, 6 = >8 times)	2.0 (1–6) *n* = 60	3.0 (1–6) *n* = 59	<0.01* (z −3.10)
11. How often have you satisfied yourself, alone, during the last month? (1 = never, 6 = > 8 times)	1.0 (1–6) *n* = 60	3.0 (1–6) *n* = 59	<0.01* (z −4.10)
12. How often do you avoid intercourse in order to avoid pain? (1 = always, 5 = never)	2.0 (1–5) *n* = 58	4.0 (1–5) *n* = 52	<0.01* (z −5.80)

*Statistically significant increase in scores

The results of Spearman’s correlation tests between the global question measured at three points in time (before, directly after and 6 months after treatment) and the total MSFQ scores are shown in Table 4. Correlation coefficients of 0.1 to 0.3 were considered to indicate a weak relationship, 0.3 to 0.5 a moderate relationship and > 0.5 a strong relationship. Correlations at all three points of time were moderate or strong.

**Table 4:** Correlation coefficents (*rho*) between the global question “*How do you feel about your sexuality and your sexual life today?”* and total scores on the MFSQ before, directly after treatment completion and 6 months after treatment

	Mean (SD)	Spearman’s correlation coefficent	*p*-value
Global question (at first consultation) and total scores for MFSQ	2.53 (2.42)	0.60	<0.01*
	38.80 (11.04)		
Global question (after completion of treatment) and total scores for MFSQ	8.96 (1.20)	0.40	<0.01*
	55.20 (14.51)		
Global question (6 months after treatment) and total scores for MFSQ	8.50 (1.80)	0.54	<0.01*
	51.90 (15.94)		

*Statistically significant correlation coefficients

Mean scores and standard deviations on the HADS sub-scales for anxiety and depression before treatment and 6 months after treatment are shown in Table 5. Results of paired samples t-tests for the anxiety sub-scale showed statistically significant decreases in anxiety levels 6 months after treatment was completed. There were no significant differences over time for scores on the depression sub-scale.

**Table 5:** Comparisons between mean scores for the sub-scales anxiety and depression on the Hospital Anxiety and Depression Scale, measured at the first consultation and 6 months after treatment using the paired samples t-test

	Total mean scores (SD)	*P-* value
Sub-scale anxiety		
Anxiety before treatment (*n* = 57)	8.77 (4.98)	<0.01*
Anxiety 6 months after treatment (*n* = 57)	7.04 (5.18)	
Sub-scale depression		
Depression before treatment (*n* = 57)	4.28 (3.75)	0.69
Depression 6 months after treatment (*n* = 57)	4.07 (4.16)	

*Statistically significant reduction in anxiety

## Discussion

It was the intention of this study to examine whether a 10-week treatment regime for women with PVD could improve their sexual interest, response and activity and reduce their pain. The results showed statistically significant improvement in ten of the 12 variables measured on the MFSQ and the improvement was sustained up to 6 months after completion of treatment. The results also showed a statistically significant reduction in anxiety measured on the HADS sub-scale, which was sustained at 6 months after the end of treatment. No effect was seen on the depression sub-scale. Validity of the 12-item MFSQ for the population in this study was demonstrated by moderate to strong correlations with the global question.

It was encouraging that the treatment regime gave positive and sustained results for the women in this study. The goals of CBT for pain and sexual dysfunction are to help women understand their genital pain as a multi-dimensional problem, which is affected by behaviors, thoughts and emotions. The aim is to modify these factors by re-direction of negative thought pathways, with a view to improving coping mechanisms and decreasing the pain experience. Alternative thoughts can result in emotional and behavioural changes. The women in this study may possibly have negative emotional feelings about sexual activity that they have been unaware of; 80 % answered that they suffered a lack of sexual desire but it is unclear whether this lack of desire preceded the onset of PVD or not. Sexual activity starts early in life and the young child quickly becomes aware of how the world around views this activity. More clues to the aetiology of PVD might be gained by understanding individuals’ experiences of admonishment when touching their genital area early in life [27]. In the present study the therapist strove to maintain an approving attitude to sexuality in general and to individual sexual preferences. It is possible, for example, that in raising the question of masturbation as a positive sexual activity, the women were given a subliminal message that this is a normaland accepted part of life. We suggest that qualitative interviews with women suffering from PVD using a Grounded Theory approach might bring further understanding of life experiences that may cause negative emotional feelings about sexuality. Grounded Theory is particularly useful when an area has earlier been sparsely researched [28] and can be used to build theory from which an instrument may be developed, in this case, to measure emotional feelings regarding sexuality.

It is also worthy of reflection that the results showed that one third of the participants had bad experiences of gynaecological examination. We have no in-depth knowledge of what these women experienced and it would in future be of value to further investigate this question in order that professionals who carry out gynaecological examinations might learn from women’s experiences.

Mena [29] discusses the impact of dyspareunia on the dynamics of the couple relationship and also how couple dynamics affect dyspareunia. Mena examines the possibility that a woman who is no longer attracted by her husband realizes that when the pain has been treated, she no longer has a legitimate reason to avoid sexual intercourse. A man who is unsure of the couple relationship can worry that when the dyspareunia problem is treated, his partner may decide to leave him. His need is that she remains “a problem”, unable to have intercourse, which means she will not stray to another partner. In the present study we found high scores for satisfaction with partners as lovers or friends at all measurement points. There were no significant changes in these variables over time, which may indicate that the problems the women in our study experienced may not originate in relationship difficulties.

Earlier research has shown advantages of multi-disciplinary treatment teams where both medical and psycho-social causes are considered [1, 3, 16]. In the present study one individual rather than a treatment team carried out the treatments, which may be a weakness in the study design or may have built up feelings of trust in the therapist. For these women, continuity of therapist may be of particular significance.

It is problematical when the therapist and the researcher are one and the same person and that the respondents are aware that the treatment is to be evaluated. This is not an optimal scenario for the objectivity required for scientific reporting. However, one of the authors (LJK) was not involved in delivery of treatment and was mainly responsible for conduct of the analyses. In this way impartiality may have been enhanced. Problems of this kind can to some extent be avoided if the person collecting the data is not the therapist.

There was no external drop out in this study; all 60 women answered the questionnaires. As the questionnaires used were the basis for dialogue between the woman and the therapist, it is maybe not surprising that they were willing to comply at the beginning of treatment and they may also have been in a state of dependency at the time they were recruited. However, their willingness to complete follow-up questionnaires 6 months after completed treatment may be interpreted as a wish to give feedback about the treatment they received.

It was gratifying to note that the participants’ levels of anxiety decreased after treatment and that this decrease was sustained at 6 months follow-up. Recent research has indicated that it may be unadvisable to total scores for the two scales incorporated in the HADS [30] and that the two sub-scales are better reported separately, as has been done in this study. The reason why the HADS was used here was because it had earlier been noted that women with PVD often reported a history of depression. However, in this cohort, there was no evidence of substantial depression.

We are not aware that there is any published literature where the MFSQ has been used for women with PVD. Our experience was that in the main the questionnaire functioned well and the relatively low internal drop-out suggests that the respondents did not find the questionnaire irrelevant or difficult. Moreover, the 12 question version used here showed satisfying correlations with a global question about the woman’s sexual life. The history questionnaire did not contain any direct question about sexual abuse, which is a weakness given the subject of this study. Important knowledge may have been missed by lack of follow-up questions regarding earlier negative sexual experiences.

Women with PVD often experience problems in finding help for their sexual difficulties because the problem is sensitive and complex and difficult to bring up with health care professionals. More research and scientific reports in this area may provide care professionals with increased knowledge and help them to feel more confident in treating these women. Alternative treatment forms such as art therapy, hypnosis, mindfulness, acupuncture and yoga are currently being assessed and will hopefully provide some evidence for the most suitable treatment for these women [1, 2, 18, 19].

It was considered advantageous in the present study to include the woman’s partner in treatment sessions. It is possible that in this way, the partner feels more of a participant in the treatment and may be more active in carrying out the “homework”. Abstinence from vaginal penetration in the early part of treatment requires that the partner is motivated to participate in the regime. It will be important in the future to collect material from a larger population to enable us to write with greater authority about our results.

## Conclusions

This study has shown promising results from a combination treatment with de-sensitization exercises and CBT for women suffering from PVD. Regular sessions and continuity of therapist may be factors which need to be taken into consideration when planning for treatment for this group of women. Inclusion of the partner in therapy may enhance the therapeutic value of the regime.
