# Erratum to: Treatment of Provoked Vulvodynia in a Swedish cohort using desensitization exercises and cognitive behavioral therapy

**DOI:** 10.1186/s12905-015-0280-4

**Published:** 2015-12-23

**Authors:** Suzanne Lindström, Linda J. Kvist

**Affiliations:** Sexology Department, Najaden Midwifery Clinic, Drottninggatan 7, Helsingborg, 252 21 Sweden; Department of Obstetrics and Gynecology, Helsingborgs Hospital, Helsingborg, 25187 Sweden; Department of Health Sciences, Faculty of Medicine, Lund University, Lund, 22100 Sweden

After publication of the original article [1] it was brought to our attention that tables two and three (here, Tables 1 and 2) contained incorrect data in question 7 and 11. The correct tables are included below. Changes in the tables do not affect the conclusions drawn in the study.

**Table 1:** Scores for all questions on the MFSQ (median and range) and comparison of individual question scores before and directly after completed treatment using Wilcoxon signed rank test (paired)

Question	MFSQ (before treatment)	MFSQ (directly after treatment)	
	Median (range)	Median (range)	*p-value*
1. Are you satisfied with the extent of your sexual life at the present moment? (1 = very dissatisfied, 7 = very satisfied)	2.0 (1–6) *n* = 60	5.0 (1–7)*n* = 59	<0.01* (z −6.50)
2. Approximately how many times, during the last month, have you thought about sex or had sexual fantasies? (1 = never, 7 = >10 times per day)	3.0 (1–6)*n* = 60	4.0 (1–7)*n* = 59	<0.01* (z −5.10)
3. Does sexual activity give you pleasure? (1 = no pleasure at all, 7 = a lot of pleasure)	4.0 (1–7) *n* = 53	6.0 (13–7) *n* = 53	<0.01* (z −5.10)
4. How often do you feel sexually excited or stimulated during sexual activity? (Example: increased pulse, increased vaginal lubrication, skin redness, quicker breathing, increased sensitivity in the skin and erogenous areas). (1 = never, 7 = every time)	4.0 (1–7) *n* = 53	6.0 (2–7) *n* = 54	<0.01* (z −4.51)
5. How often do you achieve orgasm during sexual activity? (For example increased pulse, increased vaginal lubrication, skin redness, quicker breathing, increased sensitivity in the skin and erogenous areas). (1 = never, 7 = every time)	4.0 (1–7) *n* = 53	6.0 (1–7) *n* = 54	<0.01* (z −3.85)
6. How often is vaginal dryness a problem during sexual activity? (1 = every time, 7 = never)	4.0 (1–7) *n* = 54	6.0 (2–7) *n* = 54	<0.01* (z −3.60)
7. How often is sexual intercourse painful? (1 = every time, 7 = never)	1.0 (1–7) *n* = 53	6.0 (2–7) *n* = 53	<0.01* (z −6.01)
8. Are you satisfied with your partner as a lover? (1 = very dissatisfied, 7 = very satisfied)	7.0 (2–7) *n* = 55	6.0 (1–7) *n* = 54	0.75 (z −0.32)
9. Are you satisfied with your partner as a fellow human and friend? (1 = very dissatisfied, 7 = very satisfied)	7.0 (2–7) *n* = 54	7.0 (2–7) *n* = 54	0.06 (z −1.90)
10. How often have you had sexual intercourse during the last month? (1 = never, 6 = >8 times)	2.0 (1–6) *n* = 60	3.0 (1–6) *n* = 59	<0.01* (z −4.51)
11. How often have you satisfied yourself, alone, during the last month? (1 = never, 6 = > 8 times)	1.5 (1–6) *n* = 60	4.0 (1–6) *n* = 59	<0.01* (z −5.90)
12. How often do you avoid intercourse in order to avoid pain? (1 = always, 5 = never)	2.0 (1–5) *n* = 58	4.0 (12–5) *n* = 52	<0.01* (z −5.71)

**Table 2:** Comparisson for all questions on the MFSQ (median and range) before and six weeks after completed treatment using Wilcoxon signed rank test (paired)

Question	MFSQ (before treatment)	MFSQ (six months after treatment)	
	Median (range)	Median (range)	*p*-value
1. Are you satisfied with the extent of your sexual life at the present moment? *(1 = very dissatisfied, 7 = very satisfied)*	2.0 (1–6) *n* = 60	5.0 (1–7) *n =* 60	<0.01* (z −6.71)
2. Approximately how many times, during the last month, have you thought about sex or had sexual fantasies? *(1 = never, 7 = >10 times per day)*	3.0 (1–6) *n* = 60	4.0 (1.5-7) *n* = 60	<0.01* (z −4.50)
3. Does sexual activity give you pleasure? *(1 = no pleasure at all, 7 = a lot of pleasure)*	4.0 (1–7) *n* = 53	6.0 (3–7) *n =* 54	<0.01* (z −4.60)
4. How often do you feel sexually excited or stimulated during sexual activity? *(1 = never, 7 = every time)*	4.0 (1–7) *n* = 53	6.0 (3–7) *n =* 53	<0.01* (z −5.10)
5. How often do you acheive orgasm during sexual activity? *(1 = never, 7 = every time)*	4.0 (1–7) *n* = 53	5.0 (1–7) *n* = 53	0.01* (z −2.50)
6. How often is vaginal dryness a problem during sexual activity? *(1 = every time, 7 = never)*	4.0 (1–7) *n* = 54	5.0 (1–7) *n* = 53	<0.01* (z −3.13)
7. How often is sexual intercourse painful? *(1 = every time, 7 = never)*	1.0 (1–7) *n* = 53	6.0 (1–7) *n* = 53	<0.01* (z −5.90)
8. Are you satisfied with your partner as a lover? *(1 = very dissatisfied, 7 = very satisfied)*	7.0 (2–7) *n* = 55	6.0 (2–7) *n* = 51	0.61 (z −0.61)
9. Are you satisfied with your partner as a fellow human and friend? *(1 = very dissatisfied, 7 = very satisfied)*	7.0 (2–7) *n* = 54	7.0 (1–7) *n* = 53	0.10 (z −1.71)
10. How often have you had sexual intercourse during the last month? *(1 = never, 6 = >8 times)*	2.0 (1–6) *n* = 60	3.0 (1–6) *n* = 59	<0.01* (z −3.10)
11. How often have you satisfied yourself, alone, during the last month? *(1 = never, 6 = > 8 times)*	1.5 (1–6) *n* = 60	3.0 (1–6) *n* = 59	<0.01* (z −4.10)
12. How often do you avoid intercourse in order to avoid pain? *(1 = always, 5 = never)*	2.0 (1–5) *n* = 58	4.0 (1–5) *n* = 52	<0.01* (z −5.80)

*Statistically significant increase in scores
